# Morphology and Morphometry of the Midgut in the Stingless Bee *Friesella schrottkyi* (Hymenoptera: Apidae)

**DOI:** 10.3390/insects10030073

**Published:** 2019-03-14

**Authors:** André H. Oliveira, Wagner G. Gonçalves, Kenner M. Fernandes, Marcelo S. Barcellos, Wagner M. S. Sampaio, Marcos P. Lopes, Gustavo F. Martins, José E. Serrão

**Affiliations:** 1 Department of General Biology, Universidade Federal de Viçosa, 36570-000 Viçosa, MG, Brazil; andreoliveira.ufv@gmail.com (A.H.d.O.); wagner2gufv@gmail.com (W.G.G.); kennerbio@yahoo.com.br (K.M.F.); Wmssampaiobio@hotmail.com (W.M.S.S.); marcosbiocel@ufv.br (M.P.L.); gfmartins@outlook.com (G.F.M.); 2 Instituto Federal de Mato Grosso-IFMT. Campus avançado Diamantino, 78400-970 Diamantino, MT, Brazil; marcelo7barcellos@gmail.com

**Keywords:** digestive cells, digestive tract, paracrine function

## Abstract

*Friesella schrottkyi* is a small stingless bee (3-mm long) important for agricultural and native forest pollination. This study describes the morphology and morphometry of the midgut in *F. schrottkyi* forager workers. The *F. schrottkyi* midgut presents a single-layered epithelium with digestive, regenerative and endocrine cells. The digestive cells are similar along the entire midgut length with a spherical nucleus, apex with long striated border, cytoplasmic granules in the apical region and well-developed basal labyrinth associated with mitochondria, suggesting they are multifunctional, synthesizing digestive enzymes and peritrophic matrix compounds and absorbing nutrients. Regenerative cells are located around the basal region organized in nests with some cells with a spherical nucleus. Phe-Met-Arg-Phe-NH_2_-amide (FMRFamide) positive endocrine cells are restricted to the posterior midgut region, suggesting a paracrine function in the midgut. This is the first morphological description of the *F. schrottkyi* midgut contributing to the comprehension of the digestive process of this bee.

## 1. Introduction

*Friesella schrottkyi* (Friese) is a monotypic exceedingly small neotropical stingless bee (3 mm in length) inhabiting a small region of the fragmented Atlantic rain forest of Southeast Brazil [1] with ecological and economic importance, pollinating native and cultivated plants [2,3].

Pollination occurs during food collection (nectar and pollen) in the flowers for the bee colony performed by forager workers [4]. Because these workers leave the hive, they can be exposed to toxic compounds, such as pesticides, which may affect the alimentary canal [5,6,7,8]. However, those studies have been used for the honey bee *Apis mellifera*, whereas stingless bees have different life histories, such as small colony population (*F. schrottkyi* has ca. 330 bees/nest), long development period and mass larval provisioning, which make them more susceptible to stresses than honey bees [9]. Thus, morphological data of the alimentary canal of bees are important to understand the variations between different species and the consequences of environmental contamination for these insects.

The midgut of bees is a tubular organ with a single-layered epithelium having three cell types: (i) digestive cells, which are responsible for the synthesis of digestive enzymes and nutrient absorption [10,11]; (ii) endocrine cells that synthesize hormonal peptides controlling digestion, peristalsis, diuresis and development [12,13]; and (iii) regenerative cells that play a role in the renewal of digestive and endocrine cells [14,15,16].

The midgut of bees has been studied in some species including honey bee *A. mellifera* [17,18] and in some stingless bees [13,16,19,20]. Although these studies have contributed to our understanding of some aspects of these species, there are few representatives of social bees. The honey bee, which is a model organism for studies of insect sociality, is a small clade of corbiculate bees with a single genus and few species [21]. Conversely, stingless bees are a more diverse group of corbiculate bees with a hundred species in more than 50 genera [22].

Given the agricultural and ecological importance of bees, filling these knowledge gaps is an important goal for researchers seeking to understand the factors affecting bee morphology and physiology.

This study describes the morphology and morphometry of the midgut cells of *F. schrottkyi* workers, contributing to our understanding of the digestive process of this minute and monotypic bee.

## 2. Material and Methods

### 2.1. Bees

*Friesella schrottkyi* workers returning from foraging activity were collected in two nests located at the apiary of the Federal University of Viçosa (UFV), Viçosa, state of Minas Gerais, Brazil.

### 2.2. Histology and Histochemistry

Workers (n = 15) were dissected in 125 mM NaCl and the midgut transferred to Zamboni’s fixative solution [23] for 4 h at room temperature. Afterward, the midguts were divided into anterior and posterior regions, dehydrated in a graded ethanol series of (70, 80, 90, and 95%) and embedded in historesin Leica. Three-μm-thick sections were stained with hematoxylin and eosin and analyzed with a light microscope.

Some midgut slices (two slides chosen randomly from each of the 15 workers) were submitted to the periodic acid Schiff test (P.A.S) for localization of neutral polysaccharides and glycoconjugates [24].

### 2.3. Immunofluorescence

To identify endocrine cells, the midgut from eight workers was transferred to Zamboni’s fixative solution for 30 min, washed in 0.1 M sodium phosphate buffer, pH 7.2 containing 1% Triton X-100 (PBST) and incubated with 1:400 anti-Phe-Met-Arg-Phe-NH_2_ (FMRFamide) mouse antibodies (Peninsula Lab., San Carlos, CA, USA) in PBST for 24 h at 4 °C. The samples were then washed in PBST and incubated with 1:500 anti-mouse IgG Fluorescein Isothiocyanate-conjugated (FITC) (Sigma) in PBST for 24 h at 4 °C. Whole midguts were mounted with 50% sucrose and analyzed with a fluorescence microscope.

### 2.4. Transmission Electron Microscope

*Friesella schrottkyi* workers (n = 7) were dissected in 0.1 M sodium cacodylate buffer at pH 7.2 and the midgut transferred to 2.5% glutaraldehyde solution in the same buffer for 4 h at room temperature. The samples were divided into anterior and posterior regions, washed in the buffer and post-fixed in 1% osmium tetroxide for 2 h in the dark. The pieces were washed, dehydrated in a graded ethanol series (70, 80, 90, 95, and 98%) and embedded in LR White resin. Ultrathin sections were stained with aqueous uranyl acetate for 10 min and lead citrate for 15 min and analyzed with transmission electron microscope Zeiss EM 109, at the Nucleus of Microscopy and Microanalysis of Universidade Federal de Viçosa.

### 2.5. Morphometry

Ten midguts were used for morphometric analysis tests. Each region of the midgut (anterior and posterior) was photographed in six areas with a fluorescence microscope to count the number of endocrine cells. Six slices of each midgut region were randomly photographed using a light microscope for epithelium measurements. Sections were photographed with a 40× magnification (total area = 0.414 mm^2^) and the epithelium thickness and striated border length measured using Image Pro-Plus software (Media Cybernetics Ltd., Rockville, MD, USA).

### 2.6. Statistics

The epithelium thickness and striated border length and number of endocrine cells were compared between anterior and posterior midgut regions. This data were tested for normality with the Kolmogorov–Smirnov test and submitted to analysis of variance (ANOVA) with 5% significance level.

## 3. Results

**Figure 1:**
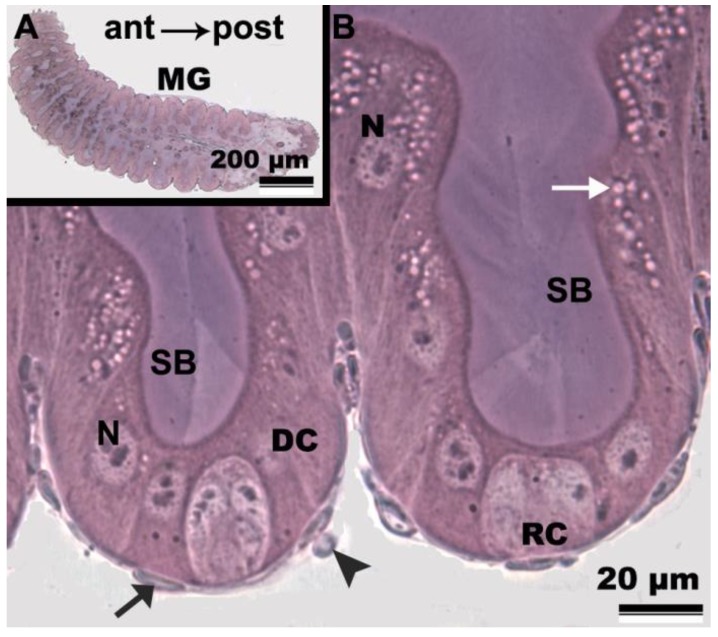
Light micrographs of the midgut of *Friesella schrottkyi* worker. (**A**) Overview of the midgut (MG). Arrow—antero-posterior orientation. (**B**) Epithelium showing columnar digestive cells (DC) with well-developed nuclei (N), apical striated borders (SB) and cytoplasm vacuoles (white arrow). Note regenerative cells nests (RC). Black arrow—nucleus of circular muscle. Arrowhead—nucleus of longitudinal muscle.

The *F. schrottkyi* midgut is a tubular organ (Figure 1A) with a single layer of columnar digestive cells having conspicuous nuclei, well-developed striated borders in their apical surfaces and two muscle layers in the basal region (Figure 1B). Nests of regenerative cells were found throughout the midgut, scattered around the base of the epithelium (Figure 1B).

**Figure 2:**
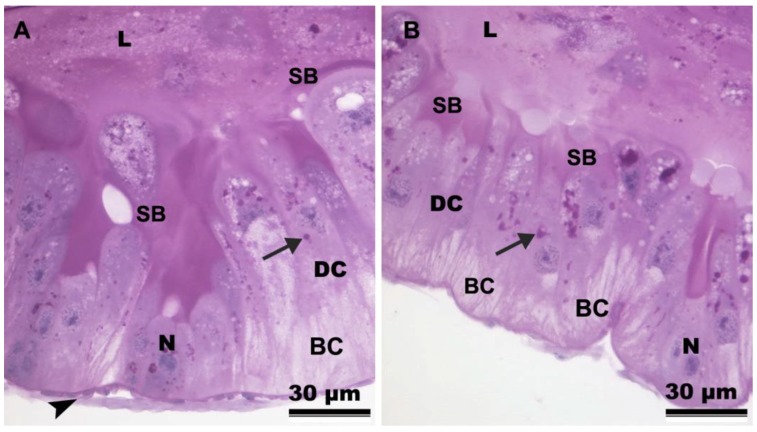
Light micrographs of the midgut of *Friesella schrottkyi* worker submitted to P.A.S histochemical test. (**A**) Anterior midgut region showing digestive cells (DC) with positive granules (arrow) in the median-apical cytoplasm and weak reaction in the basal cytoplasm (BC). Note that striated border (SB) has positive reaction. (**B**) Posterior midgut region showing digestive cells (DC) with positive granules (arrow) in the median-apical cytoplasm and weak reaction in the basal cytoplasm (BC). Arrowhead—muscle, L—midgut lumen, N—nucleus of the digestive cell.

The striated border showed stronger P.A.S.-positive reaction in the anterior midgut region (Figure 2A) when compared to the posterior one (Figure 2B). In the whole midgut, the apical cytoplasm of the digestive cells contained more P.A.S.-positive granules than the basal cytoplasm of these cells (Figure 2A,B).

**Figure 3:**
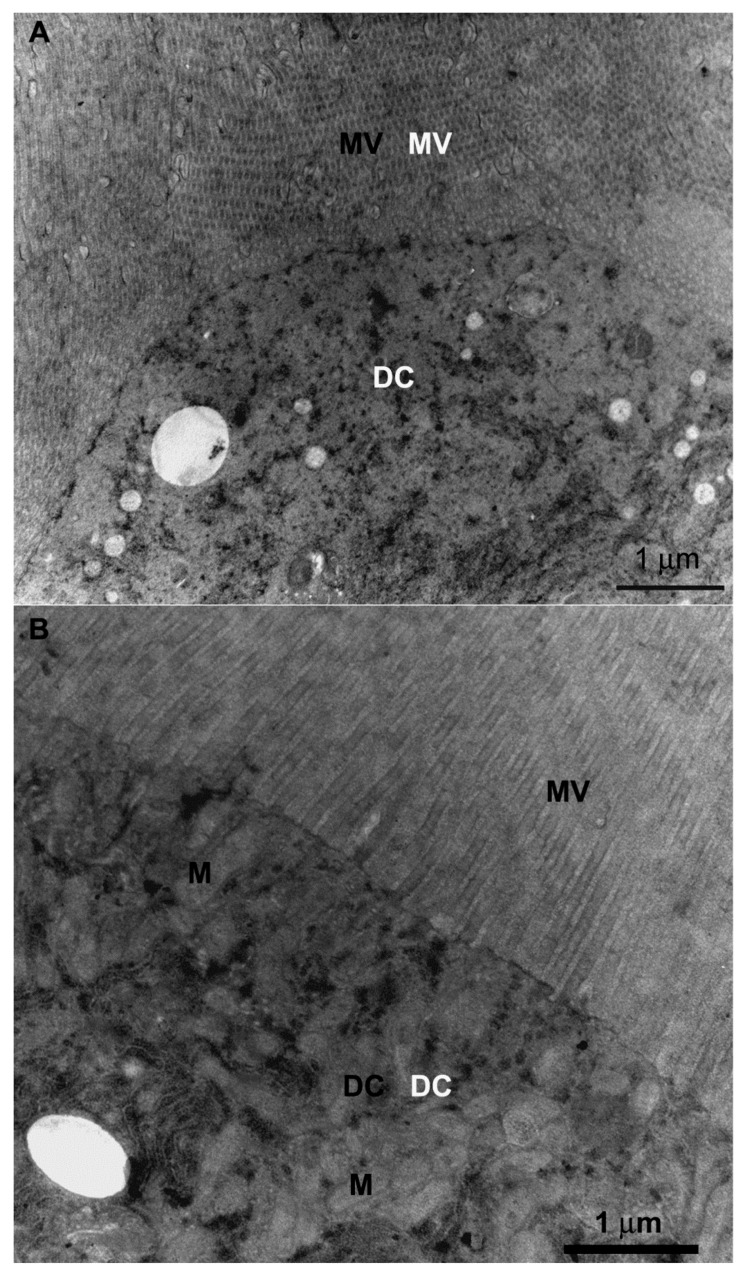
Transmission electron micrographs of digestive cell in the anterior midgut region of *Friesella schrottkyi* worker. (**A**) Apical region of the digestive cell (DC) showing many long microvilli (MV). (**B**) Apical region of the digestive cell (DC) showing closely packed microvilli (MV) and cytoplasm with mitochondria (M).

**Figure 4:**
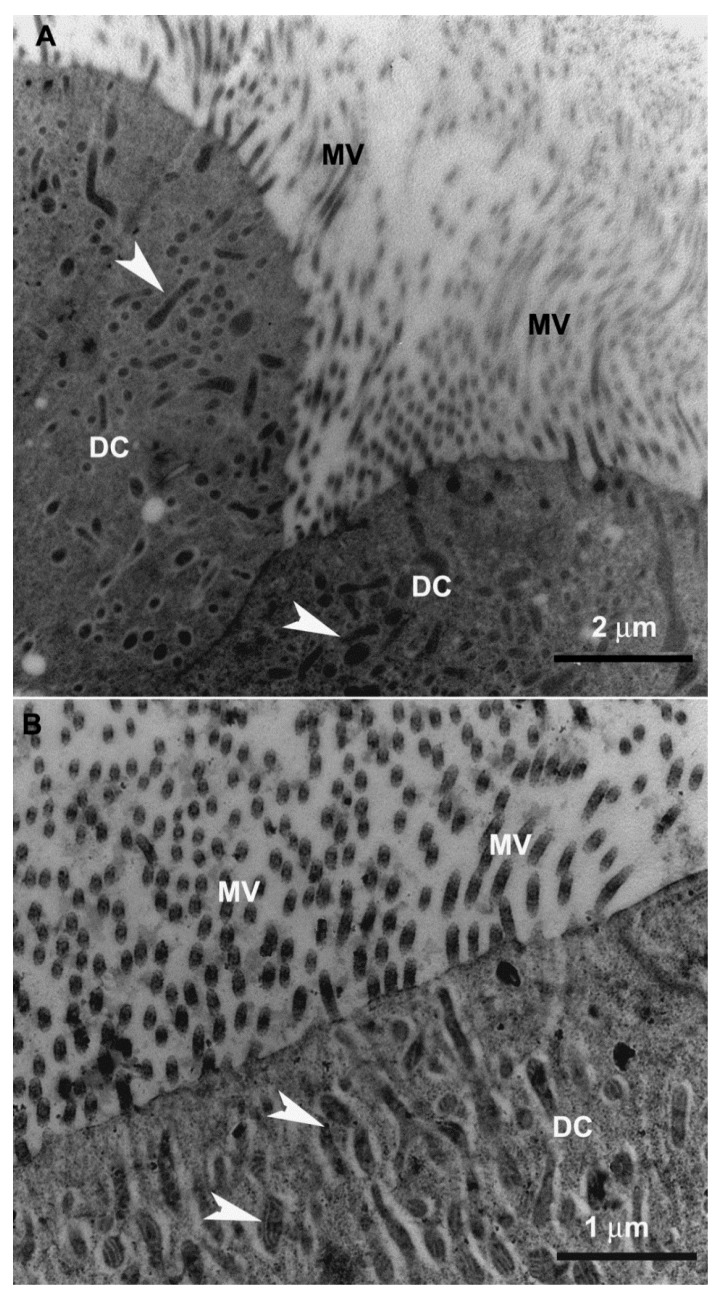
Transmission electron micrographs of digestive cell in the posterior midgut region of *Friesella schrottkyi* worker. (**A**) Apical region of the digestive cell (DC) with some long microvilli (MV) and cytoplasm with mitochondria (arrowheads). (**B**) Apical region of the digestive cell (DC) showing spaced microvilli (MV) and cytoplasm with mitochondria (arrowheads).

**Figure 5:**
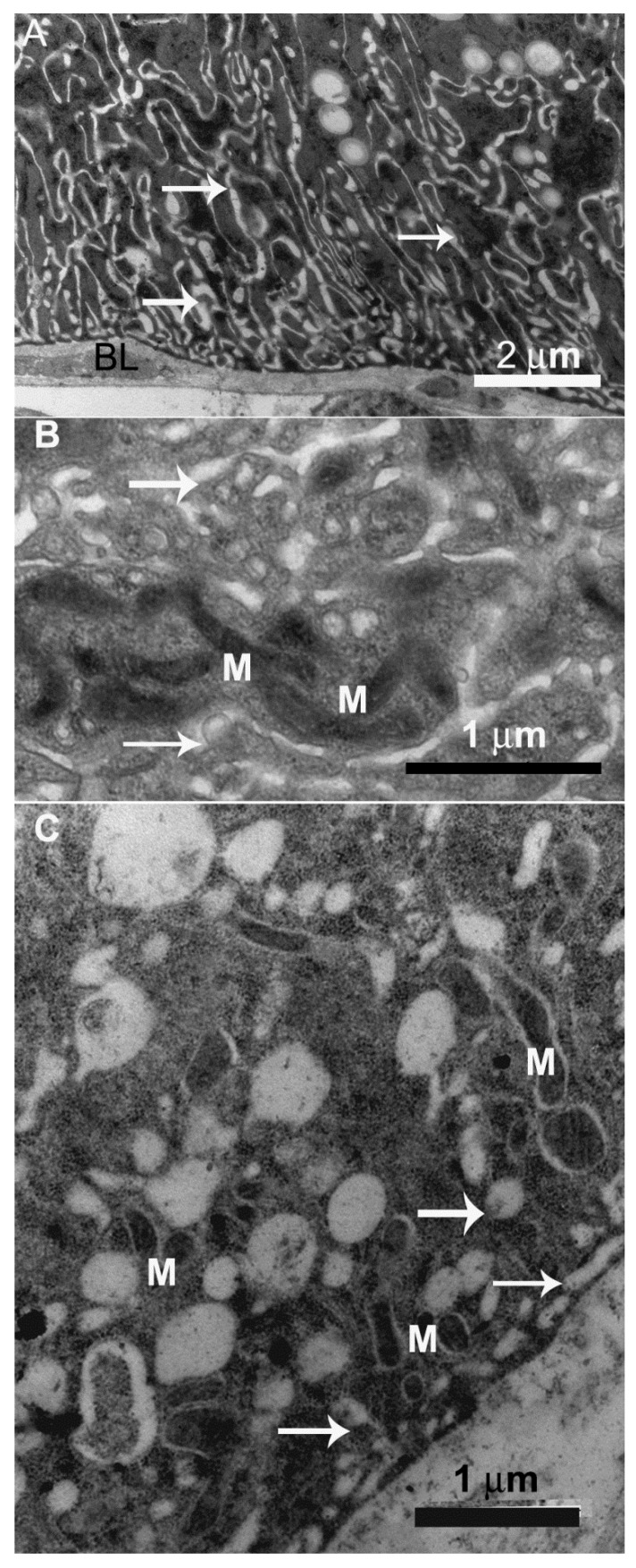
Transmission electron micrographs of digestive cell in the midgut of *Friesella schrottkyi* worker. (**A**) Basal region of the digestive cell in the anterior midgut region showing many plasma membrane infoldings forming extracellular channels (arrows). BL—basal lamina. (**B**) Details of extracellular channels (arrows) associated with mitochondria (M). (**C**) Basal region of the digestive cell in the posterior midgut region showing extracellular channels (arrows) associated with mitochondria (M).

**Figure 6:**
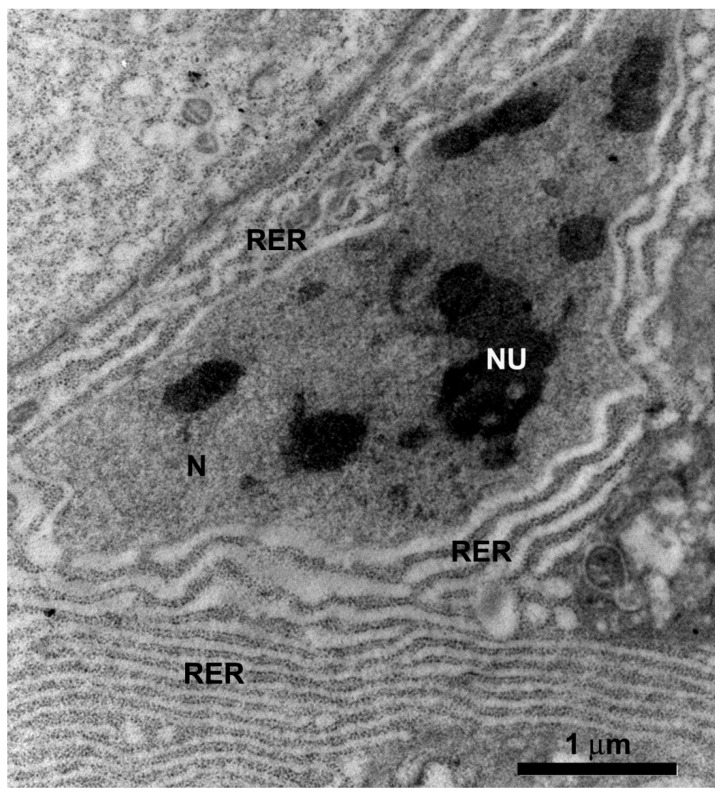
Transmission electron micrograph of the median region of the digestive cell in the midgut of *Friesella schrottkyi* worker showing well-developed nucleus (N) with decondensed chromatin and large nucleolus (Nu) and the perinuclear cytoplasm with rough endoplasmic reticulum (RER).

**Figure 7:**
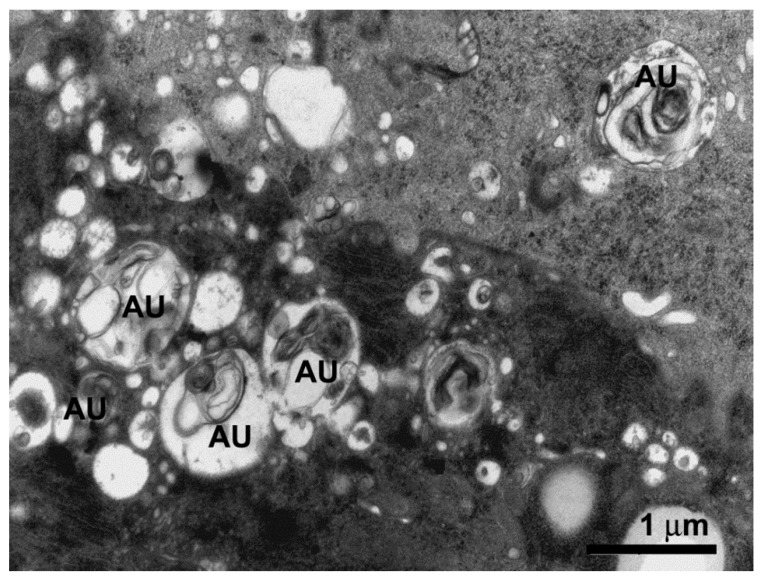
Transmission electron micrograph of median region of the digestive cell in the midgut of *Friesella schrottkyi* worker showing cytoplasm with many autophagosomes (AU) filled with membrane debris.

The digestive cells of the anterior midgut region of *F. schrottkyi* showed long closely packed microvilli (Figure 3A,B) whereas those in the posterior region were long and spaced (Figure 4A,B). In both, anterior and posterior midgut regions, the apical cytoplasm of these cells were rich in mitochondria (Figure 3A,B). The basal cell region was characterized by many basal plasma membrane infoldings (Figure 5A) associated with mitochondria in both anterior (Figure 5B) and posterior (Figure 5C) midgut regions. Along the entire midgut length, the cytoplasm of the digestive cells was rich in rough endoplasmic reticulum (Figure 6) and autophagosomes filled with membrane debris (Figure 7).

**Figure 8:**
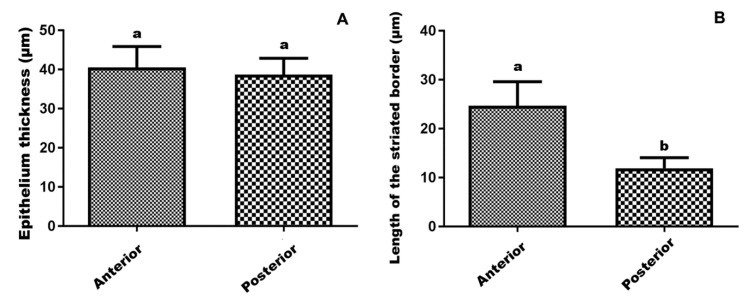
Morphometry of the midgut epithelium of *Friesella schrottkyi* worker. (**A**) Epithelium thickness (mean ± sd) in the anterior and posterior midgut regions. (**B**) Length (mean ± sd) of the striated border of the digestive cells in the anterior and posterior midgut regions. Different letters onto bars show differences between anterior and posterior midgut regions at 5% of significance level.

The morphometric data showed similar (*p* > 0.05) epithelium thickness (40 μm) between the anterior and posterior midgut regions (Figure 5). However, the striated border of the anterior midgut region was longer (23.51 μm) than that of the posterior region (11.7 μm, *p* <0.001, Figure 8).

**Figure 9:**
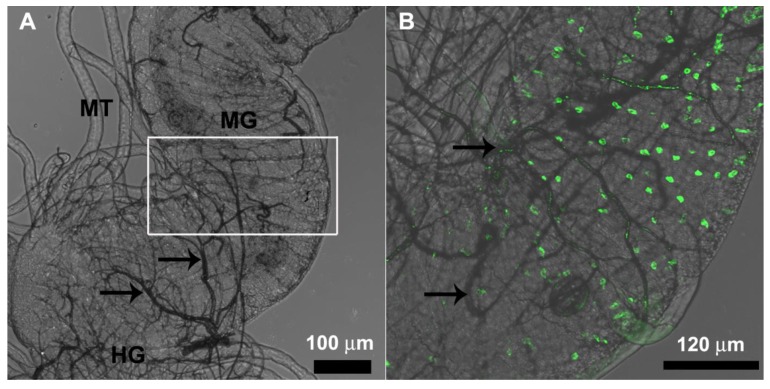
Immunofluorescence of the whole midgut of *Friesella schrottkyi* worker. (**A**) Bright field showing the posterior midgut region (MG) closely the transition to the hindgut (HG) with Malpighian tubules (MT) and some trachea (arrows). (**B**) High magnification of the square in the Figure 1A showing FMRFamide-positive endocrine cells (green) scattered in the posterior midgut region and some trachea (arrows). Merged of bright field and fluoresce images.

FMRFamide-positive endocrine cells were found only in the posterior midgut region (Figure 9A,B).

## 4. Discussion

The *F. schrottkyi* midgut is lined by a single layer of columnar digestive cells with well-developed striated border and two muscle layers showing the same pattern as other Hymenoptera [11,25,26,27].

The regenerative cells in the *F. schrottkyi* midgut are located in the basal region of the epithelium and never reach the organ lumen, similar to what is found in the bees *Trigona hypogea* [11] *Bombus morio* [27], *Apis mellifera* [28], *Melipona quadrifasciata* [16] and in the parasitoid wasp *Campoletis flavicincta* [26].

The P.A.S histochemical test shows an occurrence of positive granules in the median-apical cytoplasm of the digestive cells and in the *F. schrottkyi* midgut lumen, suggesting the secretion of glycoconjugates throughout the midgut [13,26,29], in contrast to *C. flavicincta* with glycoconjugates granules scattered throughout the whole cell [26]. These P.A.S.-positive granules may be digestive enzymes [10,20] or compounds of the peritrophic matrix that are secreted by digestive cells along the whole organ [30,31] to be released into the midgut lumen.

Mitochondria associated with the basal plasma membrane invaginations of the *F. schrottkyi* digestive cells suggest active transport of solutes, which may direct an inflow of water into the basal labyrinths [27,32]. Thus, the mitochondrial association with these enlarged labyrinths in the digestive cells along the entire *F. schrottkyi* midgut suggests that, perhaps due to the small size of the gut in this bee the whole organ plays a role in water and ion absorption, different to reports for the orchid bee *Euglossa townsendi* [33] and the bumble bee *Bombus morio* [27], which have dilated and well-developed basal labyrinths only in the anterior midgut region.

Autophagosomes in the digestive cells of *F. schrottkyi* suggest the high activity of these cells since the autophagosomes participate in the removal of damaged organelles and turnover of intracellular compounds. Digestive cells of bees have been claimed to be multifunctional synthesizing digestive enzymes [11,17,19,20,34], peritrophic matrix compounds [30,31] and membrane protein transporters for nutrient absorption [33]. All these functions demand high energy consumption via ATP generated in the mitochondria, which can lead to the production of a high level of reactive oxygen species resulting in mitochondrial damage [35] as well as endoplasmic reticulum stress [36,37], which are removed by autophagy.

*Friesella schrottkyi* presents differences in its digestive cells according to the midgut region with a longer striated border in the anterior than the posterior region, as found in the stingless bees *Trigona spinipes* and *T. hypogea* [38]. The size of the striated border is linked to the high absorption rate by digestive cells in stingless bees [38], honey bees [39], bumble bees [27] and in the Hemiptera, *Brontocoris tabidus* [40].

Our findings show that *F. schrottkyi* with its small body size (3 mm) has long microvilli (23.51 μm) in the digestive cells. However, larger bees such as *B. morio* (15.6 mm) and *T. spinipes* (7 mm) [41] present 8 μm and 2.5 μm long microvilli, respectively [11,27]. Since the size of some organs such as the alimentary canal [39] and ovaries [42] are proportional to the body size of bees, we suggest that the length of microvilli in the digestive cells of *F. schrottkyi* are longer than those of bees with a larger body size to compensate for the small size of its midgut.

An intriguing finding of this study is that microvilli of digestive cells in the anterior midgut region are longer than those in the posterior midgut region. A possible explanation might be a high level of absorption in the anterior midgut. In the midgut of insects with peritrophic matrix, such as bees, an endo-ectopertitrophic counter-current flux occurs with food moving to the posterior digestion into the endoperitrophic space and the digestion products cross the peritrophic matrix through the ectoperitrophic space and move back to the anterior midgut region due to high level of nutrient and water absorption in this region [43,44]. In addition, the main sugar source for bees is nectar that is diluted by water; a high amount of water would dilute midgut digestive enzymes. That can be mitigated by a rapid water removal by absorption in the anterior midgut, a common feature in insects feeding on diluted diets [45,46]. In fact, midgut of bees has digestive cells microvilli increase ca. 200-fold the cell surface area [38] and are rich in membrane water transporter proteins aquaporins [47]. So, the occurrence of longer microvilli in the anterior midgut region of *F. schrottkyi* may be an adaptation to that high level of absorption in this gut region.

FMRF-positive endocrine cells in *F. schrottkyi* are restricted to the posterior midgut region as observed for *B. morio* [27], *Melipona quadrifasciata* [48], *Scaptotrigona xanthotricha* [49], and solitary bees [50]. Endocrine cells have been reported to control gut peristalsis and enzyme synthesis [51]. In *S. xanthotricha*, FMRFamide-positive cells were also reported to control nutrient absorption through digestive cells [49]. The FMRF-positive endocrine cells in the posterior midgut region of *F. schrottkyi* probably control the entire midgut due to the paracrine action of their peptides, which, due to the small size of the organ, may be released by a small number of cells in a restricted midgut region. Midgut endocrine cells in bees have a paracrine function because the secretory granules release their content in the hemolymph and not in the midgut lumen [19,48].

## 5. Conclusions

The midgut of *F. schrottkyi* has digestive cells with enlarged basal labyrinths and long microvilli, suggesting morphological and physiological adaptations for nutrient absorption throughout the entire midgut length, probably due to the small size of the organ in this minute bee. Overall, our work lays the foundation for future studies evaluating the cytotoxic effects of environmental pollutants in the midgut of this non-target and important pollinator.
